# Outcome after Treosulfan-based conditioning in a real-world MDS cohort

**DOI:** 10.1038/s41409-025-02705-z

**Published:** 2025-09-09

**Authors:** Christina Rautenberg, Jacob Pyka, Tim Lohmann, Jennifer Kaivers, Annemarie Mohring, Artur Schneider, Nils Leimkühler, H. Christian Reinhardt, Judith Metzdorf, Thomas Schroeder

**Affiliations:** Department of Hematology and Stem Cell Transplantation, West German Cancer Center Essen, University Hospital Essen, Essen, Germany

**Keywords:** Myelodysplastic syndrome, Stem-cell therapies

## To the Editor:

Allogeneic stem cell transplantation (allo-SCT) is the only curative treatment for patients with myelodysplastic syndromes (MDS), offering superior survival compared to non-transplant therapies [1, 2]. However, since myelodysplastic syndromes (MDS) mainly affect older patients, the decision for allo-SCT must balance risks and benefits. Advances in transplant include the introduction of Treosulfan as reduced-toxicity conditioning, based on results from the randomized controlled (RCT) phase-III MC-FludT.14/L-trial comparing Fludarabine/Treosulfan (Flu/Treo) with reduced intensity Fludarabine/Busulfan. Here, Flu/Treo led to an improved overall survival (OS) in patients with MDS or acute myeloid leukemia (AML) due to reduced non-relapse mortality (NRM) and comparable relapse rates [3, 4]. With great interest we read the report by Stelljes et al. who performed a real-world (RW) analysis on Flu/Treo conditioning in MDS presenting comparable results among the RW setting and the RCT [5]. However, data on patient-, disease- and transplant-related factors potentially influencing outcome after Flu/Treo conditioning in MDS are limited. Therefore, this retrospective analysis aimed to evaluate RW outcomes after Flu/Treo in MDS and to analyse which patient-, disease- and transplant-related factors impact posttransplant outcome with focus on disease burden and pretransplant strategy. In-/exlusion criteria are shown in the Supplement. The study was approved by the local ethics committee (approval number: 22-10708-BO) and patients provided written informed consent. Data cut-off was November 1, 2022. Definitions, response assessment and statistical methods are described in the Supplement.

Sixty-one patients matching the inclusion criteria allografted between 2010 and 2022 were included. Ninety percent (*n* = 55) were >50 years and median age was 60 years (range 25–76). Hematopoietic cell transplant-comorbidity index (HCT-CI) was low/intermediate in 46% and high in 54% of patients. Median blast count pretransplant was 8% (range 0–17). Abnormal karyotype was present in 56% with complex karyotype in 17 patients. Based on IPSS-R, 70% had high/very high-risk disease. Regarding pretransplant strategy, 47% received upfront allo-SCT, while 46% and 7% received hypomethylating agents (HMA) or intensive chemotherapy. Among treated patients, 41% achieved complete remission (CR), while 59% had active disease pretransplant. Most patients received transplant from matched unrelated (62%), followed by matched related (16%), mismatched unrelated (15%) and haploidentical donors (7%). In-vivo T-cell depletion was performed in 79%, either using ATG or post-transplant cyclophosphamide (*n* = 9). For further analyses we dichotomized patients according to pretransplant bone marrow (BM) blast count into those with higher (≥10%, *n* = 20) and those with lower (<10%, *n* = 41) disease burden. Regarding different patient-, disease- and transplant characteristics no differences besides a higher amount of de novo compared to therapy-related MDS (t-MDS) in the group with ≥10% BM blasts were observed. Baseline characteristics are summarized in Supplementary Table 1 for the entire cohort and for patients with <10% and ≥10% BM blasts pretransplant.

**Fig. 1:**
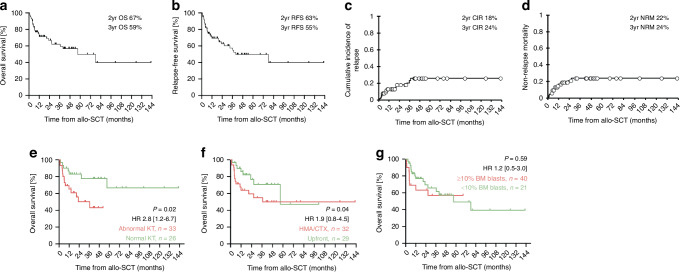
Posttransplant Outcome after Flu/Treo conditioning in MDS. This Figure depicts posttransplant outcome in terms of Overall Survival (**a**), Relapse-Free Survival (**b**), Relapse Incidence (**c**) and Non-Relapse Mortality (**d**) as well as Overall Survival depending on karyotype (**e**), pretransplant strategy (**f**) and BM blast count prior allo-SCT (**g**). BM bone marrow, CIR cumulative incidence eof relapse, CTX chemotherapy, HMA hypomethylating agents, HR hazard ratio, KT karyotype, NRM non-relapse mortality, OS overall survival, RFS relapse-free survival, yr year.

After a median follow-up of 42 months (range 1–143), median RFS and OS were 40 (range 0.9–143) and 57 months (range 1–143). The estimated 2-year RFS, OS, CIR and NRM rates were 63%, 68%, 18% and 22% (Fig. 1). Engraftment data, day +100 mortality and GvHD incidence are depicted in the Supplementary Table 2. We further analyzed impact of patient-, disease- and transplant-specific factors on outcomes after Flu/Treo. In univariable analysis, abnormal karyotype was associated with inferior RFS (*p* = 0.009, HR 3.1 [1.3–7.5]) and OS (*p* = 0.004, HR 3.7 [1.4–9.7]) and complex karyotype also negatively impacted RFS (*p* = 0.048, HR 2.1 [0.99–4.6]). Pretreatment trended towards worse RFS (*p* = 0.09, HR 1.96 [0.9–4.3]) and showed inferior OS (*p* = 0.04, HR 2.3 [0.99–5.5]) in contrast to upfront allo-SCT. In contrast, unrelated donor transplant (*p* = 0.07, HR 0.48 [0.22–1.1]) and in-vivo T-cell depletion (*p* = 0.06, HR 0.47 [0.2–1.04]) showed a trend towards improved RFS. Interestingly, BM blasts pretransplant did not affect outcome, regardless of thresholds (<5%/<10%). Additionally, older age (≥58 years) was associated with reduced relapse risk (*p* = 0.03), but also increased NRM by trend (*p* = 0.09) resulting in comparable RFS and OS to younger patients. Furthermore high HCT-CI was linked to higher NRM (*p* = 0.01), however this did not result in decreased OS (*p* = 0.1). To avoid overfitting in multivariable analysis for OS/RFS, the number of covariables was limited to three based on the amount of events observed. Based on the results from univariable analysis (*p* < 0.1) and on clinical relevance we chose karyotype, pretransplant strategy and BM blasts to be incorporated in the multivariable model. Here, elevated BM blasts (≥10%) showed no statistical significant impact on RFS/OS. In contrast abnormal karyotype and pretransplant cytoreduction clearly predicted worse OS, but not RFS. Results for univariable/multivariable analyses are depicted in Supplementary Tables 3 and 4.

In this RW analysis, we report promising outcomes following Flu/Treo in a well-characterized MDS cohort and identified abnormal cytogenetics and pretransplant cytoreduction as disease- and treatment-related factors influencing posttransplant OS. On the other hand disease burden in terms of BM blasts pretransplant had no impact.

Our RW cohort is comparable to the population in the RCT in terms of age (median 60 years in both groups) and a high HCT-CI (>3) (RW 54% vs RCT 58%) indicating a relevant risk for NRM in both populations. Accordingly, NRM was similar between the cohorts (RW 22% vs RCT 21%). While 50% in the RCT and 47% in our RW cohort received upfront transplantation, 33% of our RW patients had ≥10% BM blasts pretransplant - a variable not reported in the RCT. Furthermore, 70% of our patients exhibited a high/very high IPSS-R risk compared to 55% in the RCT, which may explain the higher relapse rate in our cohort (2-year CIR 18% vs. 11%). Consequently, 2-year RFS (63%) and OS (68%) were slightly lower compared to the RCT (68% and 73%), but still remarkable and thereby underline efficacy of Flu/Treo in high risk MDS [3–5]. In our multivariable analyses, abnormal karyotype and pre-transplant debulking was associated with lower OS after Flu/Treo, yet disease burden pretransplant reflected by BM blast count did not. This is in line with findings by Murdock et al., showing that in older AML patients transplant outcomes are determined by baseline genetics, not by disease burden [6]. Furthermore, also a recent EBMT analysis suggested no benefit of pre-transplant debulking [7]. In accordance with our results Stelljes et al. did not report a significant impact of BM blasts (<10% vs. 10–20%) on OS after Flu/Treo [5]. Interestingly, while abnormal karyotype and pretransplant cytoreduction adversely affected OS, they did not influence RFS, suggesting similar relapse rates but poorer response to salvage therapy in case of posttransplant relapse. One possible explanation is that clonal selection during pretransplant cytoreduction may result in more resistant disease at relapse. However, this hypothesis remains speculative and should be addressed in prospective studies.

Altogether, despite its retrospective design and small sample size our results suggest the following implications for clinical practice: (1) Flu/Treo conditioning is safe and effective in MDS independent of pretransplant BM blast count. (2) Pre-transplant cytoreduction has no benefit, but might even be disadvantageous. (3) The lacking impact of pretransplant disease burden on outcome after Flu/Treo challenges the common use of sequential conditioning which is largely based on retrospective data in patients with higher blast count [8] and emphasizes the need for prospective trials comparing sequential versus Flu/Treo conditioning. (4) However, also Flu/Treo seems not to be able to overcome disease-specific, non modifiable risk of adverse genetics asking for incorporation of novel approaches into conditioning.

## Supplementary information

Supplemental Material

## Data Availability

All data can be provided to researchers on individual request.
