# Supplementary material for: Outcome after Treosulfan-based conditioning in a real-world MDS cohort
Source: Bone Marrow Transplant. 2025 Sep 9;60(11):1544–6. doi: 10.1038/s41409-025-02705-z (PMC12583125; doi:10.1038/s41409-025-02705-z)
Supplement: Supplementary file 1 — Supplemental Material [file 41409_2025_2705_MOESM1_ESM.docx]

**Supplemental Material**

**Outcome after Treosulfan-based conditioning in a *real-world* MDS cohort**

Christina Rautenberg^1^, Jacob Pyka^1^, Tim Lohmann^1^, Jennifer Kaivers^1^, Annemarie Mohring^1^, Artur Schneider^1^, Nils Leimkühler^1^, H. Christian Reinhardt^1^, Judith Metzdorf^1^, Thomas Schroeder^1^

^1^Department of Hematology and Stem Cell Transplantation, West German Cancer Center Essen, University Hospital Essen, Essen, Germany

***In- and Exclusion Criteria***

Inclusion criteria were:

(1) first allogeneic stem-cell transplantation for MDS

(2) conditioning with Treosulfan 10-14 g/m² × 3 plus Fludarabine

(3) available bone marrow (BM) blast count within one month prior transplant

Exclusion criteria were:

(1) second or subsequent allogeneic stem-cell transplantation for MDS

(2) other Treosulfan-based regimens (e.g., with Thiotepa or sequential protocols)

(3) participation in the MC-FludT.14/L trial.

***Definitions and Response***

MDS subtypes were classified according to WHO 2016 and genetic risk using the IPSS-R (1). Response to pretransplant debulking therapy was assessed according to the revised International Working Group (IWG) 2023 response criteria (2). Hematopoietic cell transplantation-specific comorbidity index (HCT-CI) and graft-versus-host disease (GvHD) were defined as previously described (3–5).

1. Khoury JD, Solary E, Abla O, Akkari Y, Alaggio R, Apperley JF, u. a. The 5th edition of the World Health Organization Classification of Haematolymphoid Tumours: Myeloid and Histiocytic/Dendritic Neoplasms. Leukemia. Juli 2022;36(7):1703–19.

2. Zeidan AM, Platzbecker U, Bewersdorf JP, Stahl M, Adès L, Borate U, u. a. Consensus proposal for revised International Working Group response criteria for higher risk myelodysplastic syndromes. Blood. 1. Februar 2023;blood.2022018604.

3. Sorror ML, Sandmaier BM, Storer BE, Maris MB, Baron F, Maloney DG, u. a. Comorbidity and Disease Status–Based Risk Stratification of Outcomes Among Patients With Acute Myeloid Leukemia or Myelodysplasia Receiving Allogeneic Hematopoietic Cell Transplantation. J Clin Oncol. 20. September 2007;25(27):4246–54.

4. Glucksberg H, Storb R, Fefer A, Buckner CD, Neiman PE, Clift RA, u. a. CLINICAL MANIFESTATIONS OF GRAFT-VERSUS-HOST DISEASE IN HUMAN RECIPIENTS OF MARROW FROM HL-A-MATCHED SIBLING DONOR,S: Transplantation. Oktober 1974;18(4):295–304.

5. Filipovich AH, Weisdorf D, Pavletic S, Socie G, Wingard JR, Lee SJ, u. a. National Institutes of Health Consensus Development Project on Criteria for Clinical Trials in Chronic Graft-versus-Host Disease: I. Diagnosis and Staging Working Group Report. Biol Blood Marrow Transplant. Dezember 2005;11(12):945–56.

***Statistical Analysis***

Continuous variables were reported as medians (range) and compared with the Mann–Whitney U test. Categorical data were displayed as frequencies and assessed using Fisher’s exact test. Overall survival (OS) and relapse-free survival (RFS) were estimated with the Kaplan–Meier method and compared by log-rank test. Cumulative incidence of relapse and non-relapse mortality (NRM) were analyzed using competing risks and Gray’s test. Variables with p < 0.10 in univariate analysis or those deemed clinically relevant were included in multivariate Cox regression. Statistical analyses were performed using GraphPad Prism®, Microsoft Excel and SPSS.

Supplemental Table 1. Patient- and Transplant Characteristics for the entire cohort and for the subgroups of pts with <10% and 10-19% bone marrow blasts at transplant

Supplemental Table 2. Toxicities and Posttransplant Outcome

Supplemental Table 3. Outcome after Treosulfan-based conditioning

– univariable analysis

Supplemental Table 4. Outcome after Treosulfan-based conditioning – multivariable analysis

**Supplemental Table 1. Patient- and Transplant Characteristics for the entire cohort and tor the subgroups of pts with <10% and 10-19% bone marrow blasts at transplant**

| Characteristics | No. (%)  all pts Blasts <10% Blasts 10-19% *P*  (n=61) (n=41) (n=20) | | | |
| --- | --- | --- | --- | --- |
| Age at transplant, years (median, range) | 60  (25–76) | 60  (25-73) | 66  (31-76) | 0.36 |
| Gender  female  male | 25 (41)  36 (59) | 17 (41)  24 (59) | 8 (40)  12 (60) | >0.99 |
| Karnofsky (n=61)  ≥80  <80 | 61  - | 41 (100)  - | 20 (100)  - | >0.99 |
| HCT-CI (n=61)  low  intermediate  high | 11 (18)  17 (28)  33 (54) | 8 (20)  14 (34)  19 (46) | 3 (15)  3 (15)  14 (79) | 0.11 |
| MDS subtype (n=61)  de novo  t-MDS | 50 (82)  11 (18) | 30 (73)  11 (27) | 20 (100)  - | 0.01 |
| IPSS-R (n=56)  Very low / low  intermediate  high / very high | 2 (3)  15 (27)  39 (70) | 2 (6)  12 (33)  22 (61) | -  3 (15)  17 (85) | 0.08^a^ |
| Karyotype (n=59)  normal  abnormal  complex  non-complex | 26 (44)  33 (56)  17 (52)  16 (48) | 16 (40)  24 (60)  13 (54)  11 (46) | 10 (53)  9 (47)  4 (44)  5 (56) | 0.41^b^  0.38^c^ |
| BM blast count at diagnosis (median, range) | 7.5 (0-17) | 10 (0-16) | 12.5 (2-17) | 0.013 |
| Disease Status at transplant (n=61)  Upfront  HMA  CTX | 29 (47)  28 (46)  4 (7) | 19 (46)  19 (46)  3 (7) | 10 (50)  9 (45)  1 (5) | >0.99 |
| Time between Diagnosis and allo-SCT, months (median, range) | 6.7  (1.9-67.1) | 6.5  (1.9-62.8) | 7  (3.2-67.1) | 0.3 |
| HLA-Matching (n=61)  MUD  MRD  MMUD  haploidentical | 38 (62)  10 (16)  9 (15)  4 (7) | 25 (61)  6 (15)  8 (20)  2 (5) | 13 (65)  4 (20)  1 (5)  2 (10) | 0.52^d^  0.52^e^ |
| Donor gender  male  female | 43 (70)  18 (30) | 29 (71)  12 (29) | 14 (70)  6 (30) | >0.99 |
| CMV-Status (Donor/Recipient), n=61  pos/pos  pos/neg  neg/neg  neg/pos | 35 (57)  5 (8)  17 (28)  4 (7) | 23 (56)  4 (10)  13 (32)  1 (2) | 12 (60)  1 (5)  4 (20)  3 (15) | 0.26^f^ |
| Stem cell source (n=61)  PBSC  BM | 58 (95)  3 (5) | 39 (95)  2 (5) | 19 (95)  1 (5) | >0.99 |
| Conditioning  Treo 10 g/m^2^  Treo 12/14 g/m^2^ | 42 (69)  19 (31= | 28 (68)  13 (32) | 14 (70)  6 (40) | >0.99 |
| In vivo T-cell depletion (n=61)  yes  ATG  Pt-Cyc  no | 48 (79)  39 (81)  9 (19)  13 (21) | 34 (83)  29 (85)  5 (15)  7 (17) | 14 (70)  10 (71)  4 (29)  6 (30) | 0.32 |

^a^ IPSS-R very low/low and intermediate vs. high/very high risk, ^b^ abnormal vs. normal KT, ^c^ complex vs. non-complex KT, ^d^ HLA matched vs. mismatched donor, ^e^ related vs. unrelated donor, ^f^ recipient CMV-IgG pos vs. neg

ATG, anti-thymocyte globulin, BM, bone marrow; CTX; chemotherapy; hematopoietic cell transplantation – specific comorbidity index; HMA, hypomethylating agends; IPSS-R, international prognostic scoring system – revised version; MDS, myelodysplastic syndrome; MMUD, mismatched unrelated donor; MUD, matched unrelated donor; MRD, matched related donor; neg, negative; no., number; PBSC, peripheral blood stem cells; pos, positive, Pt-Cyc, post-transplant cyclophosphamide; tMDS, therapy-related MDS

Numbers in parentheses display patients with available information.

**Supplemental Table 2. Toxicities and Posttransplant Outcome**

| Characteristics | No. (%)  all pts Blasts 10% Blasts >10-19% *P*  (n=61) (n=41) (n=20) | | | |
| --- | --- | --- | --- | --- |
| Posttransplant Follow-Up, months (median, range) | 42.1 (1.1-143) | 47 (2.6-143) | 42 (1.1-69) | ns |
| ANC engraftment d+28^a^  yes  no | 95 | 92 | 97 | 0.56 |
| PLC engraftment d+28^b^  yes  no | 97 | 96 | 97 | >0.99 |
| time to ANC >0.5/nl, days (median, range) | 17 (9-30) | 15 (10-20) | 19 (9-19) | 0.02 |
| time PLC >20/nl, days  (median, range) | 14 (9-33) | 13 (10-32) | 14 (9-33) | 0.38 |
| acute GvHD  yes  >III°  no | 47 (78)  3 (5)  14 (23) | 30 (73)  2 (5)  11 (27) | 17 (85)  1 (5)  3 (15) | ns |
| Chronic GvHD  yes  severe  no | 32 (53)  14 (23)  28 (47) | 22 (54)  10 (24)  19 (46) | 10 (50)  4 (20)  9 (45) | ns |
| 2-yr OS (%) | 68 | 69 | 63 | 0.59 |
| 2-yr RFS (%) | 63 | 67 | 53 | 0.46 |
| 2-yr CIR (%) | 18 | 10 | 28 | 0.26 |
| 2-yr NRM (%) | 22 | 20 | 24 | 0.54 |
| d+100 mortality (%) | 5 | 0 | 7 | 0.18 |

^a^ ANC >1/nl, ^b^ PLC >20/nl

ANC, absolute neutrophil count; d, day; GvHD, graft-versus-host disease; CIR, cumulative incidence of relapse; n, number; NRM, non-relapse mortality; OS, overall survival; PLC platelet count; RFS, relapse-free survival

**Supplemental Table 3. Outcome after Treosulfan-based conditioning – univariable analysis**

| Variable | 2yr-OS (%)  % *P* | | 2yr-RFS (%)    % *P* | | 2yr-NRM (%)  % *P* | | 2yr-CIR (%)  % *P* | |
| --- | --- | --- | --- | --- | --- | --- | --- | --- |
| Gender  female  male | 63  72 | 0.75 | 57  63 | 0.69 | 34  12 | 0.15 | 12  23 | 0.65 |
| Age at diagnosis (median)  >58  <58 | 67  69 | 0.66 | 59  59 | 0.65 | 27  11 | 0.09 | 12  30 | 0.03 |
| HCT-CI  high  low/int | 51  88 | 0.10 | 45  76 | 0.18 | 34  8 | 0.01 | 19  16 | 0.61 |
| Type of MDS  t-MDS  de novo MDS | 62  69 | 0.88 | 53  61 | 0.63 | 18  36 | 0.45 | 11  19 | 0.94 |
| IPSS-R Score  High/very high  Very low/low/int | 65  66 | 0.88 | 58  56 | 0.82 | 23  17 | 0.69 | 17  27 | 0.54 |
| Karyotype  complex  non-complex | 53  79 | 0.11 | 35  65 | 0.05 | 16  48 | 0.15 | 23  13 | 0.15 |
| Karyotype  abnormal  normal | 50  83 | 0.004 | 47  69 | 0.009 | 35  10 | 0.04 | 16  21 | 0.94 |
| Disease Status  HMA/CTX  Upfront | 60  77 | 0.04 | 52  67 | 0.09 | 20  23 | 0.74 | 21  15 | 0.40 |
| Blast Count at Transplant  5-19%  <5% | 72  63 | 0.49 | 59  55 | 0.98 | 22  21 | 0.76 | 21  12 | 0.33 |
| Blast Count at Transplant  10-19%  <10% | 71  66 | 0.59 | 68  49 | 0.46 | 24  20 | 0.65 | 29  10 | 0.26 |
| HLA-matching  mismatched  matched | 67  68 | 0.55 | 57  60 | 0.45 | 20  22 | 0.99 | 23  17 | 0.54 |
| Donor  unrelated  related | 67  69 | 0.60 | 61  54 | 0.19 | 21  23 | 0.90 | 17  23 | 0.14 |
| CMV status patient  positive  negative | 71  70 | 0.56 | 63  59 | 0.94 | 19  24 | 0.92 | 11  29 | 0.46 |
| Conditioning  Treo 10 g/m^2^  Treo 12/14 g/m^2^ | 62  71 | 0.22 | 59  59 | 0.73 | 22  21 | 0.81 | 17  20 | 0.69 |
| In vivo T-cell depletion  yes  no | 63  69 | 0.25 | 55  60 | 0.18 | 20  27 | 0.64 | 18  18 | 0.23 |

allo-HCT, allogeneic hematopoietic cell transplantation; CIR, cumulative incidence of relapse; CTX, chemotherapy; HCT-CI, hematopoietic cell transplantation-specific comorbidity index; HLA, human leukocyte antigen; HMA, hypomethylating agents; HR, hazard ratio; int, intermediate; IPSS-R, Revised International Prognostic Scoring System; MDS, myelodysplastic syndrome; neg, negative; NRM, non-relapse mortality; OS, overall survival; P, p-value; pos, positive; RFS, relapse-free survival; t-MDS, therapy-related MDS

**Supplemental Table 4. Outcome after Treosulfan-based conditioning – multivariable analysis**

| Variable | OS  *P* HR, 95% CI | | RFS  *P* HR, 95% CI | |
| --- | --- | --- | --- | --- |
|  |  |  |  |  |
| Karyotype  abnormal  normal | 0.002 | 4.5  [1.7-9.7] | 0.34 | 1.8  [0.5-6.2] |
| Pretransplant Strategy  HMA/CTX  Upfront | 0.008 | 3.4  [1.4-8.5] | 0.11 | 2.9  [0.8-11.2] |
| BM Blast Count at Transplant  >10-19%  10% | 0.08 | 2.5  [0.8-6.4] | 0.09 | 2.9  [0.9-11.3] |

BM, bone marrow; CI, confidence interval; CTX, chemotherapy; HMA, hypomethylating agents; HR, hazard ratio; OS, overall survival; p, p-value; RFS, relapse-free survival
